# Research on the evolution of urban design from the perspective of public health under the background of the COVID-19

**DOI:** 10.1177/0020720921996598

**Published:** 2021-02-27

**Authors:** Hanmao Liu, Po H Wang

**Affiliations:** 1 Faculty of Civil Engineering and Architecture, Wuyi University, Wuyishan, China; 2 Faculty of Innovation and Design, City University of Macau, Macau, China

**Keywords:** Public health, urban design, isolation, quarantine, healthy city

## Abstract

There is a deep relationship between urban design and public health, and the urban built environment plays an important role in shaping human health and well-being. Globally, under the influence of the COVID-19, the interdisciplinary research between the two disciplines has once again attracted attention. From the perspective of public health, the origin of the relationship between the disease and urban design was traced, and the urban epidemic prevention in the “isolation-quarantine-epidemic prevention-traceability” epidemic was discovered response to the changing process. In response to frequent epidemics, it is proposed that urban design needs to return to a healthy city model oriented by public health and public health needs. While promoting the layout of urban epidemic prevention, it actively develops coping strategies for interdisciplinary collaborative research, in order to provide new insights and thinking for urban development research.

## Introduction

Since the outbreak of COVID-19 in 2020, in the face of the severe public health crisis, many countries have issued the “grounding” or “lockdown” laws in cities, and citizens should take self-protection measures in isolation and at home to prevent the continuous spread of the virus. Although most cities have reopened so far, the recurrence of the epidemic continues to have a number of destabilizing and lasting effects on society. The virus has revolutionized the way of life and the pace of operation in the city. As a result, the urban landscape has been changed. During the lockdown, the life of most people has been reduced to the space within the family. The usual hustle and bustle of the city was replaced by emptiness, forming a rare scene of silence. Urban public Spaces are almost always places to venture into. However, human beings have never stopped fighting against the epidemic. Although suffering has left an indelible pain on human civilization, it has also spawned a series of countermeasures and shaped the great urban civilization.^1^ The complex relationship between “disease and city” has been constantly discussed: whether urbanization and high-density urban forms accelerate the constant dispute of disease transmission;^2–3^ The relationship between infection risk and virus source among different populations was revealed through historical data reproduction,^4^ and an effective social distance scheme was proposed to simulate influenza transmission,^5–6^ or arguments on the critical view that “social alienation” inhibits social development from an anthropological perspective;^7^ There are also studies on smart city construction and epidemic disease prevention and control,^8^ architectural practice and residential environment can quickly recover social attributes,^8–10^ housing crisis can lead to potential public health problems,^11^ and pandemic risk factors that cannot be ignored in the context of sustainable development.^12^

In fact, the health of urban residents is intrinsically related to urban design and planning practice.^13^ Behind every large-scale epidemic, urban construction and development are correspondingly promoted, and the improvement of public health system and the enhancement of public environmental awareness are correspondingly promoted.^14^ But nearly half a century of rapid urbanisation and public policy seems to have weakened this link. Cities are huge complex systems that are prone to become incubators for potential viruses. Modern cities are developing too fast in pursuit of benefits to ignore the unpredictable impact that highly infectious diseases or human health problems may bring to cities. Urban development cannot be separated from the promotion of public health events, and the development of modern medicine and public health itself cannot be separated from the support of cities. They promote each other and shoulder the common mission of protecting human civilization. Stands in the Angle of the development history of the human city, to examine the determinants of human health, comb summary public health events behind the update iteration of the urban design concept and method, will find disease disaster is not only a time of crisis, but also constantly promote the healthy and sustainable development of city an important opportunity.

## Public health policies in the process of urbanization

### Urban design and public health

Cities are the global norm in the 21st century, with more than half of the world's population now living in cities, and that number is expected to rise to 70 percent by 2050. The convenient regional and even global transportation means of cities are closely connecting people in different cities. Industry, science and technology, culture and art and other elements representing the development of human civilization are all conceived and created in cities. Urbanization, as a global process, is changing the social and environmental landscape of the five continents. On another level, however, urbanization poses many challenges to global health and the pandemic.^15^ Urbanization is the result of the natural growth and migration of urban population. In a short time, excessive concentration of population in a limited space often leads to cities at the center of nodes playing the role of the source of large-scale infection and spread of disease. Multiple factors, such as excessive spatial clustering in urban forms, point-like diffusion of high-density and super-high-rise residential buildings, linear extension of large capacity and high-frequency public transportation, and deterioration of environment and sanitation, provide ways for the transmission and transmission of the disease, which are the important reasons for the rapid outbreak of the disease.

Urban design and public health share a common mission: both manage complex social systems, aim to improve human well-being, focus on the population level and rely on community-based participatory approaches, with an emphasis on needs assessment and service delivery. The emergence of modern urban planning and design has a considerable part of the important reason is to alleviate the urban management lag caused by urban population agglomeration and the ensuing public health, citizen health and a series of urban problems. Public health is also derived from the health actions taken by human beings to deal with infectious diseases. In the process of fighting against infectious diseases, personal hygiene gradually integrated into living habits and cultural customs, and public health measures became the organized behavior of the society, and later became the main means for the government to protect the health of the people. Urban civilization gave birth to the modern public health system, whose research and development have been greatly developed under the strong support of cities. In the face of increasingly frequent outbreaks of diseases in the process of urbanization, public health, as a professional discipline emerging only in modern times, is playing an increasingly important role in the prevention, control and management of urban infectious diseases. Initially, public health was primarily interested in the study of individual human models, rather than the interaction between individual health and environmental factors. The urban planning and design discipline, in contrast, focuses on geographical models of human needs and interactions within a spatial-centric framework. With the passage of time, the two disciplines are influenced by each other and learn from each other in the subsequent development process, which broadens the analytical tools and research perspectives of the disciplines. Many of the issues they study can benefit from the perspectives and methods of the other, as they share a common mission to provide a safe and healthy living environment for citizens. Today, both fields continue to be studied in depth and become increasingly important areas of research to address the threats facing cities and communities and to lead society and cities to a more efficient and equitable future.

### Public health and urban renewal

The COVID-19 outbreak is not the first time that mankind has faced a global public health emergency. In history, there have been pandemics such as plague, smallpox and malaria that have swept the world. In the 20 years since the beginning of the 21st century, mankind has experienced pandemics such as SARS, Ebola and avian flu. We don't know what the next major pandemic will be, but we do know that public health events have a long history of advancing social space, and that scientific planning systems, urban design and livable Spaces will ultimately be important weapons in the fight against disease.

Disease influence and shape the city, a number of typical urban management act and design practices in response to the outbreak of disease, such as public health crisis and development, profoundly affected the modern urban planning and design: the 17th century Europe bubonic plague pandemic, promoted the sewer system of large-scale urban planning design and construction, and develop new transition concentration zoning laws to prevent personnel, increase the risk of infection;^16^ The “urban disease” caused by the industrial revolution and rapid urbanization in Britain in the second half of the 18th century, the public health Act that was born clarified the important relationship between public health and urban design.^17^ Under the vigorous advocacy of Tang Ning and Olmsted in the 19th century, the vigorous “urban park Movement” and Howard's “garden City Theory” at the end of the 19th century in the United States were, to some extent, aimed at improving urban public health problems by increasing urban green space and other ways;^18–19^ And the influential public transport system, public housing system and modernist design inspired by tuberculosis in the early 20th century.^20^ Although the epidemic has alienated people from society and reduced unnecessary public activities, every major epidemic in history has brought us new thinking and inspiration by iterating the concept of urban design, updating people's lifestyle. In the context of major public health events in human history, a re-examination of the development process of cities, buildings and public Spaces reveals that we have “built” the defense line of human civilization by means of disease prevention and control.

## Public health events and urban design strategies

The early forms of human settlement were to conform to the natural environment, or to develop along the lakes, seas, and rivers, or to defend against external enemies. The lack of a systematic urban design; with the continuation of time, the gradually increasing human space texture and context gradually A preliminary urban system was formed; later, due to urban expansion and development, loss of control or causing public health problems, a planned urban renewal movement occurred. The pandemic has a long history in shaping urban development and promoting innovation to meet challenges. In this protracted “battle”, diseases coexist and coexist with people, affecting human evolution in complex and subtle ways and bringing disasters. At the same time as death, it also gave birth to the development of a modern public health system. The development of cities is inseparable from the progress of the public health system. The mutual game between humans and diseases has jointly shaped urban civilization.

### Isolation – The “anti-epidemic” method in early cities

**Figure 1.**
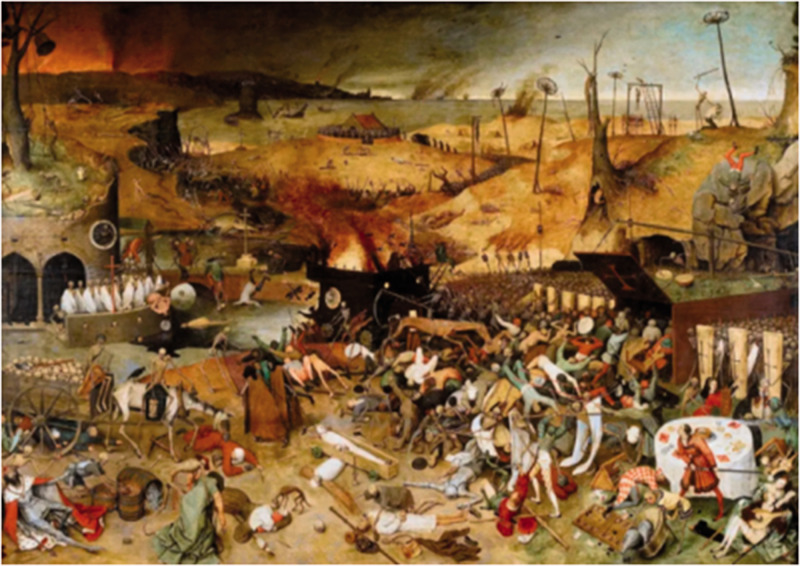
An outbreak of the Black Death in Europe in the 16th century was widely believed to be a divine punishment. The Dutch painter Pieter Bruegel the Elder wrote “The Triumph of Death” to expose the church's ideological control over people and its dark reign.

In ancient times, people often attributed the occurrence of epidemic diseases to the disasters brought by the gods of heaven. For example, during the Reign of Emperor Guangxu of qing Dynasty in China, the plague was prevalent in Macao. The worshippers of the neighborhood from Foshan in Guangdong province respectfully invited Lord Bao for a parade to suppress Wenjun.^21^ The epidemic, in the Context of medieval Europe, is interpreted as being associated with sin and as a divine punishment,^22^ which is largely documented in literary and artistic works (Figure 1). It is difficult for the public to make a scientific judgment on epidemic diseases. It is the continuous development of medicine that makes people gradually realize the relationship between epidemic diseases and public health, and public health issues also begin to get attention.

China has a long history of epidemic prevention, which predates western society. In the pre-Qin period, there was the idea of avoiding epidemic diseases and the concept of quarantine, which was also the first country to write quarantine into the law. Qin Lv “Sleep tiger qinjian” epidemic prevention and control records, “The city, the ghosts, hapen, what? And hapen to the house.” The effect is to find leprosy patients, will immediately notify the government, after the doctor diagnosed, will be sent to the quarantine and healthy people forced isolation.

**Figure 2.**
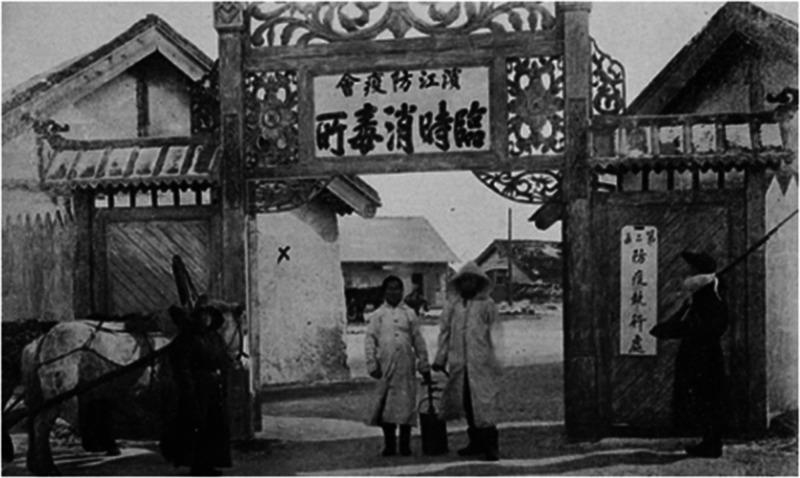
Temporary disinfection place set up for plague outbreak in Fujiadian, Harbin in The Year of Xuantong of the Qing Dynasty. Image Source: Shadow of Harbin Fujiadian Epidemic Prevention, Shanghai Commercial Press, 1911.

“Isolation ward” objectively played an important role in preventing the further spread and epidemic of leprosy,^23^ and initially formed the isolation system of infectious diseases in early ancient China. After undergoing the initial stage of the Qin Dynasty, in the Western Han Dynasty, the government “Zhuang & Cai” provided isolated houses and treatments for patients with the epidemic.^24^ In sui and Tang Dynasties, Buddhist literature records that temples were used as “isolation houses”, which were used as isolation places for malaria patients and were offered for offering and treatment.^25^ As well as the “Anji Fang” in the Southern Song Dynasty and the special leprosy hospital and disinfection house set up in the Ming and Qing Dynasties (Figure 2), the isolation methods developed in each dynasty, laying a solid foundation for the isolation and treatment mechanism in modern times.

The Advanced principles and knowledge of sanitation, Isolation and environmental sanitation were revealed in the Old Testament of western countries around the 5th century BC.^26^ It is detailed in the chapter of the book that if you have infectious disease, you should go through two rounds of 7 days for observation in isolation for a total of 14 days to determine the disease condition, and then you can leave and return to social life after the diagnosis of no infection.^27^ During the Byzantine Epidemic of Constantinople in the 6th century, the state enacted racially discriminatory laws against groups deemed responsible for the disease, Then The scholar comments,^28^ “The quarantine enacted by Justinian proved virtually useless and did nothing to stop The spread of The plague. Or, it still qualifies as a quarantine technology, A failed technology, but a technology nonetheless.” By the 12th century, similar institutions under the jurisdiction of the church had been established in European countries, and the life of patients in isolation had become a social system running separately.^29^ After centuries of leprosy and black death continues to wreak havoc in continental Europe, under the rule of the church around the shoulder the task of the isolated monastery, built a large number of individual patients home, people also gradually realize the importance of maintaining the public health, from clean air, sewage disposal, and food items such as health Angle control of the disease,^30^ but to the world, not to control the spread of the epidemic.

As you can see, the Chinese and western in treatment of epidemic control and isolation of space are improved with The Times change and development, but the scientists found that microbes before is the actual cause of the disease, early humans were lack of effective medical treatment and scientific research, isolation of patients only moderately effective, does not contain the development from the roots. This period of urban design and related space layout planning system of public health security considerations, most of epidemic prevention means starting from the practice, summarizes the method to isolate as the core to control the disease to spread, is not able to distinguish between healthy people and infected patient, from the side shows this is just a method to disease resistance of the accumulation of experience, rather than a breakthrough of research and theory.

### Disease prevention and control – Effective means of epidemic prevention in cities

#### Birth of quarantine system

Although isolation is one of the oldest and most effective health measures developed by human beings, the rapid development of cities has been unable to solve all the epidemiological problems.^31^ Drawing on previous experience on infectious diseases, the medieval society observed the correlation between time and onset of disease, and pointed out that if no disease appeared after a period of isolation, it would not be infectious.^32^ This method was also called quarantine. The word quarantino comes from the Italian “Quarantino,” meaning 40 days in quarantine.^33^ The concept of quarantine is rooted in health practices.^34^ However, unlike quarantine, quarantine is aimed at exposed people who are not sick. It requires a clearer understanding of the causes and ways of disease transmission.^28^

In order to protect coastal cities from the frequent plague in continental Europe in the 14th century, the Italian government stipulated that ships and passengers arriving in Venice must stay on the nearby Island of SAN Lazaro for 40 days until a special health committee allowed them to enter the city. In 1377, Ragusa, a Venetian trading colony, passed the World's first quarantine law, the Plague Foci Act,^35^ which required that any ships and caravans arriving from an infected area must be quarantined for a month in a nearby designated town or island before they could enter the city. People began to realize that isolation was only screening out infectious diseases and that there was no treatment for those who might be exposed. From this phase of isolation, we can see that the early quarantine system is gradually taking shape.

**Figure 3.**
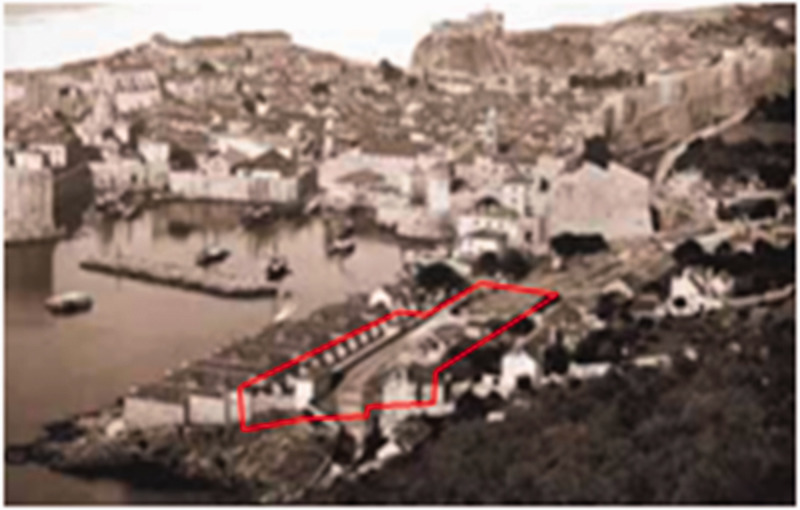
Lazaretto quarantine complex.

**Figure 4.**
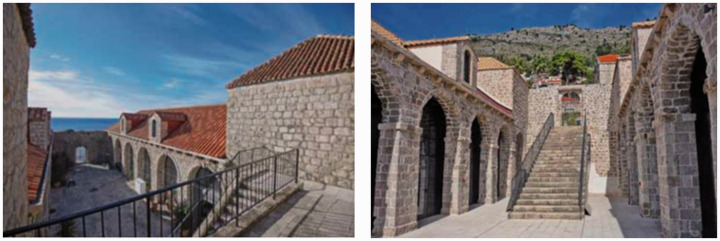
Lazaretto spatial layout design. Image Source: Lazaretto in Dubrovnik.

In the process of fighting against the recurrent epidemic, the single mode of isolation has seriously affected the urban development and has been unable to meet the needs of urban development. The recurrence of the epidemic has become the biggest threat to public health and urban development in Lagoosa. In order to control the epidemic and ensure the operation of the city, the government built Lazaretto comprehensive quarantine complex in two phases in 1627,^36–37^ isolate and restrict the movement of people exposed to infectious diseases to see if they are ill. This is one of the few epidemic prevention institutions in history designed specifically for quarantine (Figure 3), and many historians believe that the establishment of this institution may have made an important contribution to the gradual elimination of mass plagues in Europe.^38^ The building has two major functions: medical treatment and isolation. In the event of an outbreak, as an important quarantine institution in the city, it serves the quarantine and isolation of local residents and commodity traders. It will isolate and restrict the movement of asymptomatic infected persons who have come into contact with the source of infection or asymptomatic infected persons, so as to observe whether they are ill. First of all, the design focuses on the location layout, close to the gate entrance and close to the port pier, with the purpose of facilitating the flow of merchants and travelers from the epidemic area at the source, and protecting the health of the residents in the city. Secondly, in view of the fact that the high walls in the city are easy to cause disease transmission, multiple spacious courtyards will connect the isolation rooms in series (Figure 4) to facilitate the air flow and circulation inside the building. In addition, the design takes into account the decontamination site of the cargo rather than being used solely for medical and quarantine purposes.

Different from the early cities at the expense of normal “running” in return for city brief peaceful way, lazaletto quarantine orgnaization is quarantine and become the precious heritage of integration with the design, it has cleared all potential pathogen carriers from the crowd, slow and control the outbreak of the disease, and the goods after disinfection the normal flow of trade, and fully guarantee the normal operation of the urban economic activity, realizes from experience in isolation to practice the important breakthrough of quarantine inspection. Epidemic regulations, quarantine institutions and urban control measures effectively reduced the infection rate and quickly restored urban order during the outbreak of the epidemic. Since then, this public health policy has gradually prevailed in Europe for 300 years, and its important role has been influential to this day.

#### Leonardo Da Vinci's design concept of “anti-epidemic city”

The Western Renaissance marked Europe's transition from the Middle Ages to modernity, and was hailed as an era of amazing progress in the field of art and architecture. However, when the architects of the early Renaissance paid too much attention to beauty and classical order, the thoughts and theories of the ideal city by Onardo da Vinci had gone beyond the traditional aesthetics. People rarely notice that the Renaissance in the 15th century also marked the birth of the city as a real discipline, and the important driving force behind it-frequent public health events. The plague raided Milan, Italy at the end of the 15th century. In order to reduce the risk of the spread of the epidemic, Leonardo rethinked the planning and design of medieval cities, and proposed a visionary vision of the “ideal city”: public health depends not only on disease. The cure of Neo-Confucianism is more dependent on urban ecology, environment and transportation organizations.^39^ He deeply realized that the inefficiency of the streets in the Middle Ages was the source of disease. Diseases spread through unhygienic environments and were more likely to occur in densely populated areas. The health of citizens is related to the health of their urban environment. It is necessary to make cities like Milan crowded and dirty Dirty, difficult-to-travel medieval cities have been transformed into modern cities that emphasize Renaissance aesthetics, cleanliness and high efficiency.

**Figure 5.**
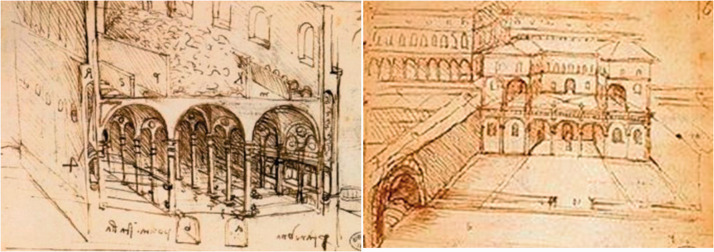
The manuscript of the vertical urban space designed by Leonardo da Vinci. Image source: French National Museum "Paris Manuscript B".

Da Vinci’s city divides different functional buildings on three levels, and connects the three functional levels through a river network as a transportation system (Figure 5): The open upper level of the city carries public life, and people can be undisturbed Walking between elegant palaces and streets;^40^ the middle layer is the space for services, commerce, transportation, and industry; the closed lower layer is the urban river system, responsible for the loading and unloading of goods and wastewater of wheeled traffic.^41^ Not only that, public health is the core element of urban design. In order to facilitate population evacuation and control the spread of the epidemic, Da Vinci proposed a number of pioneering designs: for the first time the urban volume limits the living scale of 10,000 people and the multi-residential community model To ensure that all houses have good ventilation and sunshine patterns; public toilets should be set up in residential and commercial areas, and air circulation should be ensured; urban squares should be appropriately controlled to not only meet the standards of human behavior and activities, but also to control the crowd Gathering scale; the width of the city street should be at least the same as the height of the buildings on both sides to ensure that all houses can get sufficient direct lighting and reduce the risk of epidemics after the earthquake; the street surface requirements are higher than the drainage ditches to ensure that rainwater will not be retained The pavement retains germs and drains into the underground pipeline; the water supply network and hydraulic pump system controlled by the gate have pioneered the solution of the water supply and sewage problems of occupied sidewalks, and ensured and improved the city's public water sanitation conditions.

A city that “operates” in different ways, Leonardo’s Healthy City outlines a modern and rational city plan that conforms to the ideals of the Renaissance. It shows the close relationship between urban design and disease prevention and control, and has a significant impact on the future of the city. The development of "structure" has had an important impact. The planning and design with public health as the core creatively changed the urban health status and epidemic problems caused by crowding. From today's perspective, many of Da Vinci's assumptions are not only correct, but also predictively point out solutions and ideas for public health problems that may be encountered in the process of urbanization. Although the design could not be realized due to cost and other reasons, the plan showed important changes and breakthroughs from governance to prevention, from quarantine practices to modern urban design concepts.

#### Disease map – A good recipe for the city to cure the epidemic

**Figure 6.**
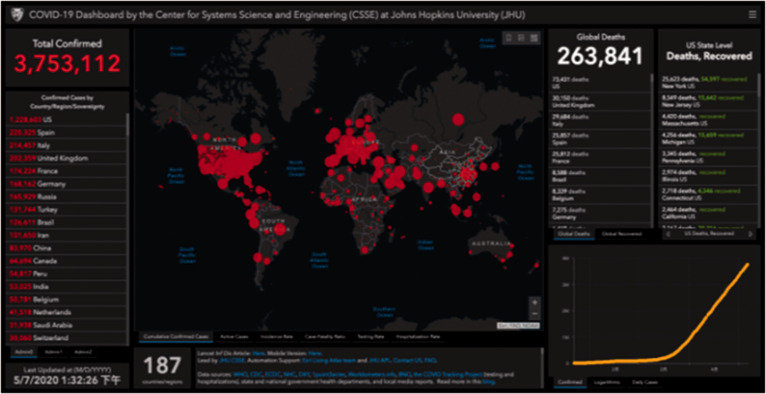
Interactive information map constructed by Johns Hopkins University to track viruses. Image source: Wellcome Collection online archives.

The mapping of disease incidence and prevalence has long been part of modern public health, epidemiological and human disease research. Disease analysis maps are being presented in more complex forms than ever before (Figure 6), helping us understand how diseases spread and optimize urban design. The spatial distribution and statistical analysis of modern epidemics have been realized because of the scientific recognition that diseases such as typhoid fever and cholera are caused by microorganisms. The spatial distribution map used to test diseases demonstrates the relationship between health and geography, as well as the relationship between specific locations of occurrence and local suspected infectious diseases.

**Figure 7.**
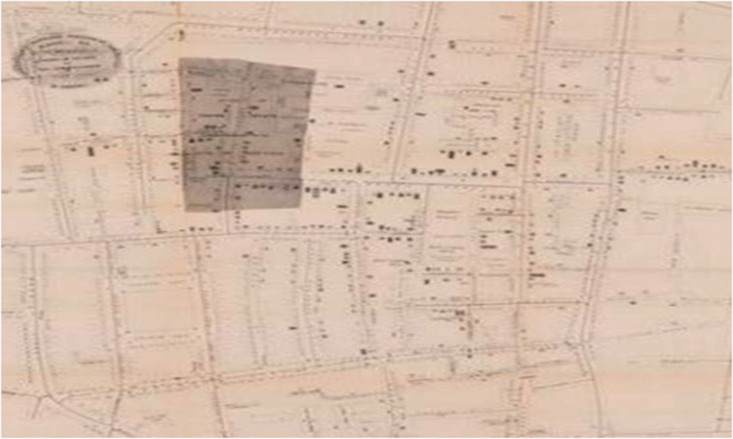
Map of Cholera in London, England in 1854. Image source: https://coronavirus.jhu.edu/map.html

In the 1850 s, when cholera broke out in London, England, doctor John Snow proposed a data science method to solve the pandemic. He used the London city map and water supply system data to draw a famous cholera map. He showed the correlation between water intake points, confirmed cases and the virus through the visualization of cholera epidemic maps,^41^ and found and confirmed conclusive evidence that cholera began to spread through contaminated drinking water (Figure 7), prompting the government authorities to launch the urban water supply system transformation plan. Victoria’s precautionary construction was realized. The modern sewage treatment system was far away from drinking water sources and the sewage was safely transported to downstream areas. Finally, the epidemic was successfully controlled by changing the community water supply mode. As a classic example of comprehensive disciplinary research methods, the epidemic has also become a turning point in modern urban design. It gave birth to the first public health act in human history, the Public Health Act of 1848, which clarified the close connection between public health and urban design. It marks the arrival of the modern public health era; secondly, the establishment of modern urban design and infrastructure construction rules guided by public health needs, and the urgency of incorporating public health into urban planning and design, is also directly reflected in Howard’s “Pastoral City” theory.

**Figure 8.**
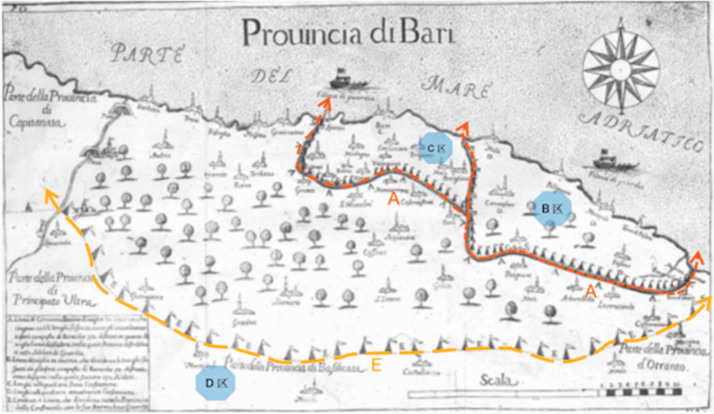
Map of the spread of the Bari plague drawn by Filippo Arrieta. Image source: The author adapted from the New York Academy of Medicine.

Before that, the first epidemic map in history, which was ignored by most scholars, could be traced back to Italy in the late 17th century,^42^ which was older than the cholera map of 19th century London. The map emerged as a means of health protection policy in the event of an epidemic and was developed and innovated over the following centuries. When the plague are spreading across Europe, Italy royal auditors filippo aleko tower (Fillippo Arrieta) mapped the Barry province plague spread to southeast Italy map, visualization to curb the spread of the disease for the first time on the space strategy,^43^ he put forward A available in cities and more areas within the scope of implementation of isolation scheme (Figure 8): military isolation boundary map identifies the “A” and “E”, respectively “B”, “C” two plague area and adjacent “D” area separated matera province. Zone B is the most severely affected, while Zone C is relatively light and under control earlier, but the two are still under strict quarantine (the icon triangle tent represents the troops on lockdown mission, and the density of tents indicates the degree of isolation). “E” is wider than “A” and extends to the whole province, reflecting the classification of the exclusion zone. The pattern of isolation on the map clearly reflects the spatial pattern of network spread that the disease outbreak presents, showing how to deploy a complex, epidemic-level isolation plan within a defined area.

Scientific progress has enabled the single infectious disease research to integrate multiple disciplines such as public health, urban design and geography, so as to have a comprehensive and scientific traceability analysis and understanding of diseases. Through the means of planning, design and renovation of basic facilities, the level of urban public health and the quality of housing are constantly improved. Under the background of urban public health needs “disease drawings”, intuitive expression from a huge amount of urban designers help planning to get more valuable information in the information, show the thinking of a new virus and virus community, the relationship between human hosts and the spread of the virus space environment, for the current public health diseases traceability screening, geographic information system (GIS) tracking the disease prevention and control of scientific means such as provides the understanding, analysis, and intervention possible development trend of public health events, has played a positive role for disease prevention and control decision.

## Urban design thinking in the “post-epidemic” period

The “black swan” of the epidemic has yet to fly far, and the future orientation of urban construction has once again become a hot topic in various fields. COVID-19 can spread to any city and region in the world in a short period of time, which shows that while the effect of urban agglomeration brings comprehensive development, innovation and interconnection, it can also push cities to the edge of crisis within minutes and seconds. When pandemics are frequent and major epidemic disasters are sweeping the world, the urban development thinking that gave priority to economy and efficiency has become vulnerable, and new rules are being formed. It is important to reassess and consider the relationship between urban development and public health. significance.

### Building a healthy city model

Urban planning and design is a discipline based on history and current situation, responding to future urban development trends, and making people's lives better. In the past few years, the discipline has paid more attention to the layout of physical space and the configuration of related facilities. There has been a tendency of “heavy things over people” and “emphasis on construction and operation”.^44^ This sudden epidemic has exposed many problems in urban governance. As an important part of the modernization of national governance, planning and design should play a corresponding role in urban governance, and work with the whole society to move from passive “crisis response” to active “crisis prevention”.

Healthy city is a brand-new development model facing the future of mankind, and it has a symbolic guiding role for the development of modern cities. Build a healthy city model, integrate health concepts with urban planning and design practices, and return urban design to its original intention of focusing on public health and taking public health as the orientation. Healthy cities emphasize: 1) The city is guided by a healthy ecological base, supported by healthy infrastructure, and encourages respect for the environment, an organic layout, and an urban development model that promotes symbiosis and mutual prosperity. 2) Healthy cities should focus on public health in all aspects from urban planning, construction to management, and integrate public health concepts into land and space planning, and integrate them with planning practices to meet the needs of the public for healthy living and work, and become a society The development of a healthy population, a healthy environment and a healthy society necessary for development as a whole. 3) Healthy cities should form a circle with public health as the core and based on community life. In response to the outbreak of infectious diseases and the growth of chronic diseases, various facilities, resources and work that promote health should be integrated to form an efficient and high-quality health governance model.

### Promote the layout of urban epidemic prevention

Compared with the several pandemics in human history, the similarities of this epidemic are mostly in large and medium-sized cities with dense living and frequent business transactions. The most significant difference is the speed and breadth of spread in time and space. The rapid spread of the new coronavirus in a short period of time has largely exposed the excessive concentration of population and resources brought about by urbanization, and the negative effects of various agglomeration phenomena at different spatial scales from the global to the local.

Before the epidemic crisis, we should rethink how to effectively promote the opportunity of healthy city planning and management, and actively respond to the problems discovered in the process of epidemic prevention and control. Look for trends and adjust methods to adapt to the “new normal” environment created by the virus, and help cities respond quickly and effectively to epidemic threats: 1) At the spatial planning level, pay attention to the dense layout of urban texture and balance the life between urban and rural areas Ways: Enhance urban resilience and resilience, improve the ability of cities to respond to sudden crises, and be able to respond quickly, recover and adapt, dynamic feedback, and maintain development in the face of major crises. 2) At the functional layout level, refer to the design of disaster prevention and refuge sites, reserve land and facilities for public health and epidemic prevention, do a good job in site selection planning, reserve traffic and infrastructure access conditions, so that it can be quickly opened in emergency situations; Integrate more urban open spaces into the core of public spaces and planned cities, and improve infrastructure construction to help implement urban emergency response and evacuation. 3) At the space management level, urban space, as an urban governance tool, maintains the operation of social relations. Modern urban governance requires big data methods. The Internet of Things is used to establish a “perception system” to accurately capture key information so as to share and monitor epidemics in real time on a smart city platform Risk and trend analysis; secondly, in response to the need for epidemic prevention and control, the types and levels of urban space should be reorganized, the space use should be optimized, and the public health monitoring and emergency response facilities should be added to form a dynamic management network.

### Carry out interdisciplinary collaborative research

Although experts in the field of public health, urban planners, and architects have coordinated to promote the development of science and technology and economic prosperity, and urban living conditions have been greatly improved, but the rapid urbanization has gradually created gaps in the cooperation between these disciplines. Close contact is one of the important reasons why we continue to face epidemic challenges today. The disconnect between public health and urban planning and design occurred at the turn of this century, a period of unprecedented development for cities. In many areas, the speed of rural population migration to cities has exceeded the ability of governments and planners to respond to people’s needs. This has led to the proliferation of informal urban settlements and the health and safety risks caused by improper mixed use of land. These will become ideal hotbeds for the occurrence of new infectious diseases and epidemics.

A healthy city requires the reorganization of urban design and public health, giving full play to the strengths of their respective disciplines, and filling each other’s vacancies,^45^ which can be implemented at the following levels: 1) In the field of discipline education, cross-curricular courses can be opened to complement each other's knowledge, or increase Urban design and public health dual-discipline joint training professional degree program to deal with the future urban public health crisis and more complex and changeable urban environment. 2) In terms of project practice, the design process will increase the subject interaction with public health, scientifically and systematically improve the comfort of physical space, and enhance the environmental quality of social public places. 3) At the level of regulations and policies, public health professionals can serve on the urban planning committee, participate in the formulation of urban planning policies, and incorporate public health into the decision-making scope of land use planning, transportation planning, and urban design. Urban planners and designers can also contribute to health The Health Committee advises and contributes to the decision-making of public health events.

## Conclusion

Great challenges can bring great innovation and creativity. The epidemic continues to spread, posing new problems and challenges to modern cities and traditional design concepts. In the face of the increasingly complex urban environment, it is of great value and significance to build a healthy city model to respond positively to future urban development trends, to promote the construction of urban epidemic prevention layout in the process of urbanization, to allow interdisciplinary research to bridge the cooperation gap behind the urbanization process, and to reconsider and weigh the relationship between future urban development and public health.We should firmly believe that realize, is the essence of human society in the face of infectious disease epidemic, the government and the society as the unit, organized, rational, scientific manner defense, each time the challenge and past behind and no essence is different, the city will eventually through continuously improve and perfect to adapt to new social order, and become more beautiful and strong.
